# What determines the success or failure of policy experiments?—A qualitative field study of China's healthcare reform pilots

**DOI:** 10.3389/fpubh.2025.1748984

**Published:** 2026-01-29

**Authors:** Xiao Yang, Guang Yang

**Affiliations:** 1 School of Labor Relations, Shandong Management University, Jinan, China; 2 School of Public Administration, Shandong Normal University, Jinan, China

**Keywords:** China, grounded theory, healthcare reform, policy experiment, the County Medical Service Community (CMSC)

## Abstract

**Background:**

Policy experimentation has become a central strategy for governments seeking to navigate complexity in healthcare reform. In China, extensive rounds of county-level healthcare reform pilots aim to strengthen primary care and improve service equity. However, while some pilots expand successfully, others stagnate or are suspended. The underlying mechanisms that generate these divergent outcomes remain insufficiently examined.

**Objectives:**

This study investigates how healthcare reform pilots evolve in practice by identifying the key actors involved in pilot management, analyzing the driving forces that shape pilot performance, and explaining the different developmental trajectories of pilots within China's county medical service community (CMSC) reform.

**Methods:**

Drawing on large-scale fieldwork across 23 CMSCs, we collected qualitative data through semi-structured interviews and focus group discussions with 127 internal and external stakeholders. Using grounded theory, the data were analyzed through open, axial, and selective coding, and subsequently integrated to construct a typological framework explaining variations in policy pilot outcomes.

**Results:**

Drawing on the coding framework, the analysis identifies two overarching forces shaping CMSC pilot implementation: institutional compatibility and policy potential energy. Institutional compatibility reflects the alignment of CMSC policy design, healthcare insurance payment rules, interdepartmental coordination, incentive structures, and resource–benefit allocation. Policy potential energy derives from the redistribution of administrative power, political momentum from higher-level governments, the capacity of policy entrepreneurs, and the contextual foundations of social capital and trust. Mapping these dynamics reveals four pilot trajectories: upscaling, adjustment, suspension, and autonomy.

## Introduction

In recent years, there has been renewed interest among governance and policy scholars in policy experiments and pilot initiatives (1–3). A growing body of research highlights that public administration and governance processes are characterized by complexity, uncertainty, ambiguity, and unanticipated consequences, and that traditional administrative approaches often fail to produce satisfactory outcomes (4–7). Many scholars argue that policy experiments play a critical role in generating policy-relevant knowledge, enabling early assessment of potential impacts, and identifying broader societal or professional implications of new policy initiatives when prior knowledge is limited (8–10). Consequently, policy experimentation has emerged as a viable, replicable, and low-cost strategy for addressing complex and wicked problems in public governance.

These challenges are particularly prominent in healthcare systems. In China, healthcare reform has undergone multiple phases of experimentation. Entering the twenty-first century, the health component of China's social safety net faced increasing inequities, as improvements in health outcomes did not automatically accompany economic growth. The urban–rural divide widened, reflecting disparities in income and healthcare utilization (11). Since 2009, the Chinese government has introduced a new round of healthcare reforms aimed at exploring reform options, improving medical services, safeguarding public health, and addressing imbalances in healthcare resources and service quality, with a broader goal of constructing a more equitable and sustainable healthcare system (11–16). Although reforms span areas such as basic medical insurance, public hospital reform, essential medicines, and service pricing, strengthening primary care has been the central strategy. By increasing resources at the grassroots level and expanding basic medical security, China aims to enhance service utilization and improve overall population health (17, 18). As reforms deepen, opportunities have emerged to build a more integrated and collaborative primary care system.

Within this evolving context, policy experimentation has become increasingly important for healthcare governance, particularly concerning how high-impact social policies can be effectively implemented. While existing research has examined pilot selection, evaluation, and diffusion in healthcare reform, relatively little attention has been devoted to the management of pilots during the experimentation process itself. Pilot management involves complex collective action dynamics, including the interaction of institutions, stakeholders, and inter-organizational relationships. Despite its importance for determining policy experiment outcomes, this process remains underexplored. To address this gap, this study adopts a systematic qualitative research design to examine China's county-level integrated healthcare reform pilots. We conducted multi-site fieldwork across 23 counties, including on-site observations and in-depth interviews with key stakeholders such as government officials, healthcare administrators, and frontline medical personnel. During the data analysis phase, grounded theory techniques were employed for open, axial, and selective coding to inductively identify core concepts and relational mechanisms. In addition, typological analysis was conducted to categorize and interpret divergent pilot trajectories. This combined analytical strategy enables a comprehensive examination of the mechanisms shaping pilot management in China's primary healthcare reforms.

This study addresses three research questions: (1) Who are the key actors involved in managing pilot programs, and how do they influence the experimental process? (2) What critical factors determine the success or failure of these pilots, and what mechanisms facilitate the scaling up of successful pilots? (3) What are the final outcomes of pilots that do not progress beyond the experimental stage? By exploring these questions, we aim to elucidate the complex dynamics of healthcare reform policy experiments and contribute to broader theoretical discussions on policy implementation in complex institutional environments.

This study makes several contributions to the literature on policy experimentation and implementation. First, it identifies and examines the roles of core actors in pilot management, illuminating how they influence pilot progression and prospects for upscaling. Second, it uncovers key driving forces that shape pilot performance, offering insights into the mechanisms underlying successful policy diffusion. Third, it develops a comprehensive typology of pilot outcomes, including an unprecedented examination of the trajectories of pilots that do not advance to full implementation. Finally, the study extends policy implementation theory and collective action theory by identifying the institutional elements and contextual factors central to healthcare reform experiments. These findings deepen our understanding of the dynamics of policy experimentation and provide practical implications for policymakers engaged in healthcare reform initiatives.

## Literature review

### Policy experimentation and piloting

There are currently two main understandings of policy experimentation. The first derives from a narrow methodological perspective, which views policy experimentation primarily as scientific research method (19). From this standpoint, policy experimentation is regarded as an evidence-generating approach that emphasizes the design and implementation of experiments to evaluate the impacts and effects of specific policies. Under this interpretation, policy experimentation requires formulating clear hypotheses, collecting data, conducting controlled experiments or observational studies, and analyzing results to provide empirical evidence for policymaking. Charles Peirce conceptualized experimentation as a scientific mode of inquiry aimed at establishing causal relationships, advocating active intervention, randomization, and statistical analysis—principles consistent with Bacon's ideal of experimental science (20).

The second perspective adopts a broader view by situating experimentation within the domain of national governance. From this angle, policy experimentation is embedded in the policymaking and implementation processes of a nation and serves to address socioeconomic problems, improve public services, and promote social development. Here, policy experimentation extends beyond data collection and effect validation to encompass the exploration of new governance models, the tackling of emerging societal challenges, and the facilitation of institutional innovation. John Dewey emphasized the practical value of experimentation in governance, understanding it as a process of testing new approaches through practice and iteratively refining them through trial and error (21). Building on this tradition, Sebastian Heilmann incorporated China's central–local dynamics into the analysis of policy experimentation, proposing the concept of “experimentation under hierarchy” as a distinctive mechanism of Chinese governance (22). These two understandings reflect different methodological orientations toward policy innovation and improvement and help clarify how scholars conceptualize and apply experimentation in theory and practice. Importantly, these perspectives are not mutually exclusive but rather complementary.

Policy experimentation in China's healthcare reform embodies both scientific inquiry and governance practice. Drawing from existing research and China's governance experience, this study conceptualizes policy experimentation in healthcare reform as a systematic and integrated process. Within this context, piloting functions as a core strategy that runs throughout the entire reform cycle, constituting a complex governance process. This process requires considering multiple factors, including the policy environment, economic development of pilot cities, and central–local relations. Each stage—pilot selection, pilot management, pilot evaluation, and pilot scaling—represents an intricate governance process of its own. As the central vehicle of experimental governance, piloting performs multiple governance functions (3). While incorporating elements of scientific research, China's policy experimentation in healthcare reform is fundamentally embedded in national governance arrangements. The pilot management process is, in essence, the policy experimentation process, reflecting a central government strategy of policy trial-and-error to assess the effectiveness of policy innovations. It constitutes a governance practice grounded in intergovernmental relations.

### Policy implementation in policy experimentation and piloting

Policy implementation theory has been a cornerstone for understanding the complex processes that unfold between policy formulation and policy outcomes. The foundations of this field were laid by Pressman and Wildavsky (23), who emphasized the numerous challenges involved in translating policy intentions into practice. Their work initiated a substantial body of research that has expanded over the past five decades, leading to diverse models and frameworks for analyzing policy implementation.

A central strand of this literature is the top–down vs. bottom–up debate. The top-down perspective, advanced by scholars such as Mazmanian and Sabatier (24), highlights the importance of clear policy goals, sufficient resources, and effective communication from policymakers to implementers. In contrast, the bottom–up approach, championed by Lipsky (25), underscores the discretion and influence of street-level bureaucrats and local actors in shaping policy outcomes. Building on these perspectives, (26) proposed the ambiguity–conflict model, which identifies four types of implementation processes based on the levels of ambiguity and conflict: administrative implementation (low ambiguity, low conflict), political implementation (low ambiguity, high conflict), experimental implementation (high ambiguity, low conflict), and symbolic implementation (high ambiguity, high conflict) (26). This framework offers valuable insights into the varied dynamics of implementation across different policy contexts.

The ambiguity–conflict model is particularly relevant to policy experiments and pilots, which typically involve high levels of ambiguity due to their innovative and exploratory nature. Experimental implementation—characterized by high ambiguity and low conflict—allows for learning and adaptation as implementers navigate uncertain and evolving conditions. This perspective aligns with the broader concept of policy learning, which has become increasingly prominent in studies of policy experimentation (27). Another key aspect of implementation theory is the recognition that implementation is a complex, multi-actor process. (28) emphasizes the critical role of inter-organizational relationships and networks in shaping implementation outcomes (28).

### Perspectives on experimentalist governance, policy learning, and the drivers of policy experimentation

A growing body of comparative public policy research provides important perspectives for understanding policy experimentation not merely as an implementation arrangement, but as part of broader experimentalist governance architectures. In this literature, experimentation is conceptualized as a governance mode that enables states to operate under uncertainty through iterative goal-setting, open-ended local discretion, systematic monitoring, and recursive revision of rules (29). Rather than relying on hierarchical compliance, experimentalist governance emphasizes distributed problem-solving capacity across multiple levels of government, creating institutional environments that support continuous learning and adjustment. This framework has been empirically validated across policy domains such as environmental regulation, social welfare, education, and digital governance in the EU, OECD countries, and Latin America.

Complementing this perspective, scholarship on policy learning highlights the cognitive, political, and organizational mechanisms through which governments absorb lessons from pilot initiatives. Policy learning research identifies distinct pathways—instrumental learning, social learning, and political learning—that shape how policymakers interpret evidence, update beliefs, and adjust governance arrangements (27, 30). Recent studies further demonstrate that learning is not simply a technical process of evidence accumulation but is embedded in power structures, professional networks, and institutional routines that influence how pilot experiences travel across jurisdictions (31, 32).

A particularly relevant strand concerns the drivers of policy experimentation, which extends the focus beyond uncertainty and problem complexity to examine the underlying motivational logics that prompt governments to initiate and sustain experimental reforms. Early accounts tended to view experimentation primarily as a response to technical ambiguity or rapidly evolving policy environments, but more recent comparative studies reveal a more diverse set of political, administrative, institutional, and cognitive incentives. Politically, governments may employ experimentation as a strategy for managing reform risks, containing distributional conflict, or signaling responsiveness while retaining flexibility under uncertain conditions, as demonstrated in analyses of climate and environmental governance experiments (33, 34). Administratively, experimentation is often driven by capacity asymmetries and fragmented authority structures that require differentiated solutions across jurisdictions; pilots create protected spaces in which officials can mobilize localized expertise, explore alternative coordination models, and compensate for organizational constraints (35, 36).

At the institutional level, experimentation may serve as a mechanism for circumventing entrenched routines, overcoming path dependence, and relaxing rigid regulatory or bureaucratic structures that would otherwise inhibit policy innovation, a dynamic identified in studies of regulatory reform and governance learning (37). Finally, a substantial body of work highlights learning-oriented drivers, arguing that experimentation facilitates iterative feedback, reflexive planning, and adaptive problem-solving in complex policy systems; such mechanisms have been observed in energy transitions, environmental management, and water governance, where pilots generate incremental knowledge that informs redesign and scaling (38, 39). Together, these studies demonstrate that policy experimentation is not merely a technocratic response to uncertainty but a multifaceted governance strategy shaped by the interplay of political incentives, administrative capacities, institutional constraints, and learning needs.

These studies underscore that experimentation does not emerge solely from top-down design but is sustained by multi-level incentives, inter-organizational relations, and the political economy of reform. Importantly, they also show that experimental governance can function in highly diverse political environments, provided that mechanisms for iteration, learning, and coordination are institutionalized.

### Background and piloting process of the county medical service community (CMSCs)

**Figure 1:**
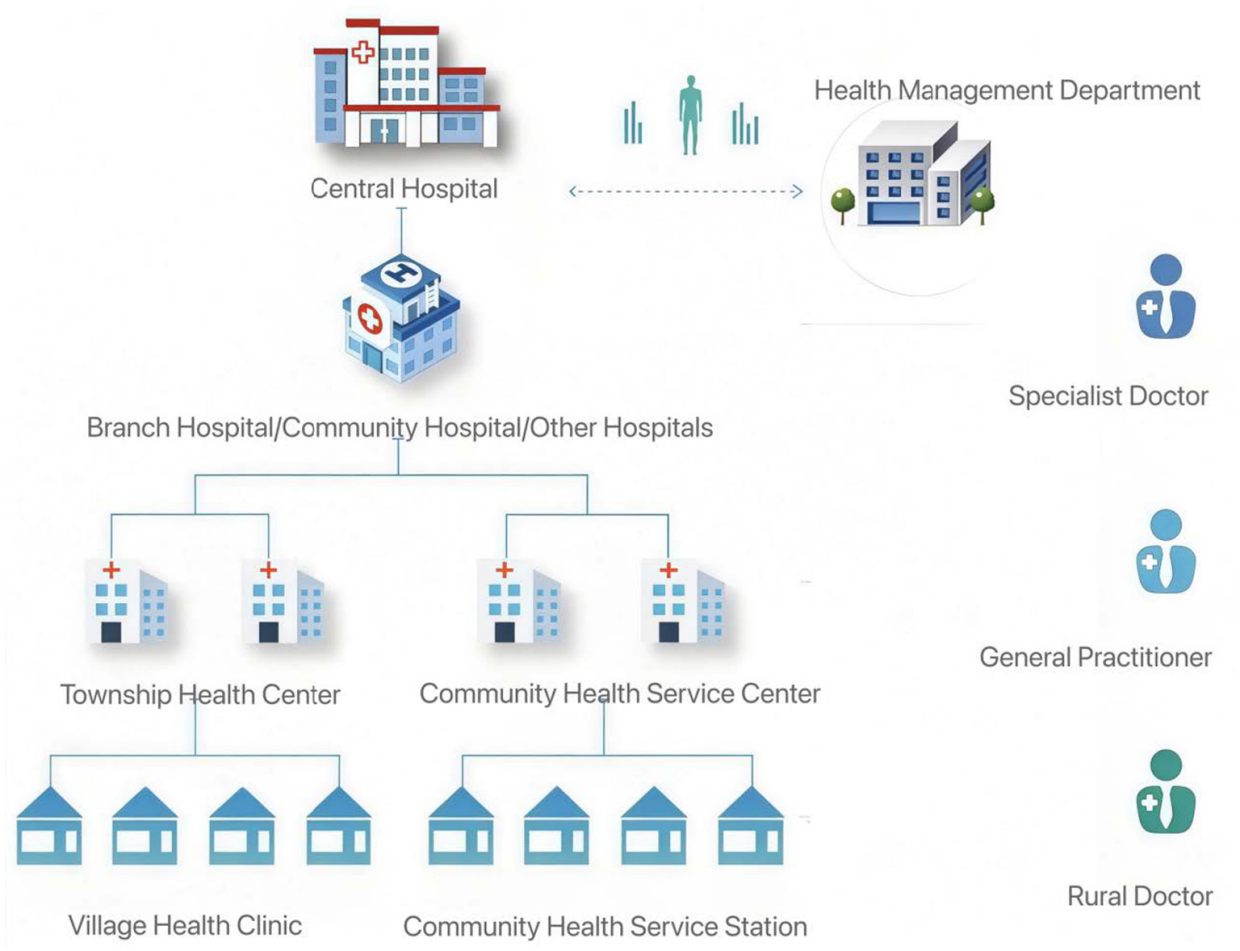
The organizational structure of the CMSC.

China's healthcare reforms have increasingly emphasized county-level integrated delivery systems as a strategy to strengthen primary care and improve system efficiency (40). Among these initiatives, the County Medical Service Community (CMSC) has become a central institutional arrangement for restructuring rural healthcare governance. The County Medical Service Community (CMSC) is a tightly integrated, county-level healthcare delivery system led by a county hospital and formed through the consolidation of county- and township-level medical and health resources. Through unified management, resource sharing, and the downward transfer of technical capacity, the CMSC establishes a community of shared responsibility, services, interests, and governance. Its overarching goals are to strengthen county-level service capacity, revitalize township-level institutions, stabilize village-level services, enhance vertical coordination, and achieve effective information integration. The CMSC operates as a single legal entity, while medical institutions within it may no longer maintain independent legal-person status, although their core functional orientation remains unchanged. Non-profit private medical institutions are permitted to join the CMSC. Rather than functioning merely as an administrative coordination mechanism, the CMSC represents a comprehensive reorganization of county-level healthcare institutions into a unified delivery and management system led by a county hospital. This reform aims to overcome longstanding problems of resource fragmentation, weak primary care capacity, and misaligned incentives across county, township, and village providers (41). Figure 1 illustrates the organizational structure of the CMSC.

The CMSC differs from earlier medical alliances in that it establishes an integrated governance structure under which county hospitals, township health centers, and village clinics operate with unified management of administration, personnel, finance, clinical standards, pharmaceuticals and equipment, and information systems (“six-unifications”). This tight integration is intended to support the national goal of “county-level strength, township vitality, and village-level stability,” thereby ensuring that common and frequently occurring diseases can be diagnosed and treated within the county.

Several institutional mechanisms are particularly important for understanding the operation of CMSC pilots and are central to the analysis in this study. First, fiscal management reforms allow CMSCs to pool medical insurance funds at the county level and adopt global budget payment with retention of surpluses. This mechanism enhances the flexibility of fund utilization and provides financial incentives for cost control and service restructuring. Second, the county medical insurance bureau plays a pivotal governance role by designing payment policies, supervising the use of medical insurance funds, and promoting coordination across medical services, medical insurance, and pharmaceuticals. Third, greater hospital autonomy—particularly in staffing, professional title evaluation, and income distribution—enables the leading county hospital to exercise effective managerial authority within the CMSC and motivates providers to participate in integrated care delivery (40, 42, 43).

These institutional arrangements distinguish the CMSC from looser regional medical alliances. Whereas, medical alliances focus primarily on service cooperation, CMSCs resemble group-based organizational restructuring, with unified legal representation and integrated management across all participating institutions. The aim is to form a cohesive organizational entity capable of reallocating resources, standardizing service quality, and facilitating coordinated service delivery across levels of care.

The CMSC reform is also accompanied by a set of evaluation dimensions—including integration strength, service homogenization, functional division, efficiency improvement, and health outcomes—that guide local implementation and assess progress. Indicators such as the proportion of primary-level visits within the county, the share of medical insurance expenditures retained within the county, and measures of chronic disease management reflect policy expectations for CMSCs to promote orderly care-seeking, enhance service quality at the grassroots level, and improve the efficiency of resource use. These metrics further highlight the policy emphasis on strengthening county-level governance capacity and improving the performance of primary healthcare institutions.

As a national policy experiment, the CMSC initiative demonstrates substantial diversity across regions, shaped by differences in fiscal capacity, health insurance policies, administrative arrangements, provider capabilities, and population health needs. This diversity underscores the importance of studying the management of CMSC pilots, especially the institutional and inter-organizational dynamics that influence their implementation trajectories. Understanding how fiscal mechanisms, insurance policies, and organizational authority interact in practice is crucial for assessing the effectiveness and scalability of CMSC reforms. These governance processes form the core focus of this study's empirical analysis.

## Research design

### Research method

Grounded Theory, anchored in the naturalistic research paradigm, emphasizes the discovery of patterns and concepts directly from empirical data and the construction of theory through an iterative process of comparative analysis and data integration (44). Rather than beginning with predetermined hypotheses, Grounded Theory relies on continuous interaction between data collection and analysis, enabling researchers to inductively derive theoretical insights from the field. In this study, Grounded Theory is applied to explore the dynamics of pilot management and policy implementation within healthcare reform experiments, using county medical service community (CMSC) pilots as empirical cases. Through focus group discussions and semi-structured interviews with key stakeholders—including health commission officials, medical insurance administrators, county hospital directors, township health center managers, and village physicians—the research investigates governance logic, operational mechanisms, constraints, and reform trajectories within CMSC pilots. The interviews covered topics such as the introduction and operation of CMSCs, organizational and financial mechanisms, policy coordination, implementation challenges, and possible solutions (45).

### Data resource

This study forms part of the national research project “Progress, Issues, Causes, Mechanisms, and Pathways of Hierarchical Medical Treatment,” commissioned by the National Healthcare Security Administration. Field investigations were conducted in 23 CMSC pilot counties. To ensure conceptual depth and maximum institutional variation, sampling considered three criteria through a theoretical sampling strategy consistent with Grounded Theory: regional representativeness, covering eastern, central, western, and northeastern China; policy relevance, incorporating both national and provincial CMSC pilots with diverse integration models; and institutional accessibility, based on counties that formally approved the field study. In addition, three focus group discussions were conducted with representatives from health administrative agencies, county hospitals, township health centers, village clinics, and medical insurance departments, aiming to triangulate perspectives and verify preliminary findings.

Before entering each field site, the research team submitted formal written research request letters to county health commissions and medical insurance bureaus. These agencies issued written approvals, which serve as institutional-level consent for the field investigation. At the individual level, participants provided verbal informed consent, which was audio-recorded before each interview. All interview transcripts were subsequently anonymized. Please refer to Tables 1, 2 for specific survey counties and interview personnel details.

**Table 1:** Regional distribution and number of interviewed stakeholders from the CMSCs.

**Region**	**The provincial-level administrative region**	**The number of CMSCs**	**Stakeholders interviewed**	**The proportion it accounts for**
Interviews at the national level	Staff members of related Administration; Media staff	/	6	4.9%
Eastern region	J province; S province; H province; A province; Z Province; E province; I Province	11	50	39.3%
Central region	U province; N province	5	20	15.7%
Western region	X province; Q province; Y province	5	43	33.8%
Northeastern region	M province	2	8	6.3%

**Table 2:** Regional distribution and number of interviewed stakeholders from the CMSCs.

**Respondents' departmental affiliations**	**Respondents' occupational roles**	**Stakeholders interviewed**	**The proportion it accounts for**
County Committee General Office	Member of the County Standing Committee, Deputy County Head in Charge	3	2.4%
Health Department	The Director, Deputy Directors, Director of the General Office, and Heads of Relevant Divisions of the Health Commission, etc.	16	12.6%
Medical Insurance Department	The Director, Deputy Directors, Director of the General Office, and Heads of Relevant Divisions of the Medical Insurance Bureau, etc.	23	18.2%
Hospitals of Different Levels within the CMSCs	The Party Secretary, President, Vice Presidents, President's Assistants, Director of the County Medical Community General Office, Heads of Departments, and Physicians of the Hospital, etc.	66	51.9%
University	Professors, Associate Professors, Lecturers, Researchers, etc.	16	12.5%
Department of Media and News	The County Health Communication Platform, Employees of People's Daily Online, etc.	3	2.4%

### Research process

The coding and analysis followed classical Grounded Theory procedures—open, axial, and selective coding—while incorporating additional steps to ensure analytic rigor. Coding was conducted manually by four doctoral researchers, each trained in qualitative analysis and health policy research. To strengthen the validity and contextual accuracy of the coding system, two collaborators from county medical insurance departments participated in several rounds of discussion, helping refine category boundaries and clarify policy-specific terminology. No qualitative analysis software was used; this decision was intentional. Given the frequent need for iterative adjustment of codes and the involvement of practitioners who were unfamiliar with specialized software, manual collaborative coding facilitated more flexible and efficient coordination, especially when interpreting policy-embedded concepts.

To ensure consistency, the four coders held weekly joint coding meetings to discuss newly coded materials, harmonize interpretations, and refine category definitions. When disagreements arose, the coding team returned to the original transcript, reviewed the contextual meaning, and collectively revised the categorization until conceptual clarity was achieved. All adjustments were documented in iterative versions of the coding manual. Approximately 20% of the transcripts were double-coded, and divergences were resolved through structured discussions, ensuring that the coders and collaborators shared a consistent understanding of category properties and coding rules.

Theoretical saturation was assessed throughout the data collection and analysis processes. Saturation was first observed during fieldwork in the 18th county, when no new concepts or properties emerged from additional interviews. To further validate saturation, the research team conducted supplementary interviews across the final five regions. The 5 interviews conducted after the point of preliminary saturation—covering county hospitals, township health centers, village clinics, health commissions, and medical insurance bureaus—did not generate new categories or alter the conceptual structure.

Prior to formal coding, all interview materials were processed using two layers of labeling to ensure clarity, traceability, and analytic rigor. First, each interview record was assigned a unique identification code composed of the interview location, the date of the interview, and the initials of the interviewee. For example, the code DQ20230615WXL indicates that the interview took place in location DQ on June 15, 2023, with WXL as the interviewee. Second, to strengthen coding transparency and facilitate precise referencing, the transcript was sequentially numbered on a sentence-by-sentence basis. These detailed identifiers combined the interviewee code with paragraph and sentence numbers, enabling analysts to accurately locate and verify each coded segment during the analytic process.

## Result

### Open coding

Open coding, also known as first-level coding, requires coders to maintain neutrality and openness, comprehensively capturing key information from the interview content. Similar information is abstracted and labeled, forming initial categories. In this study, raw interview data from 127 internal and external stakeholders of the county medical service communities were organized by question or theme into phenomenon definitions. Identical or similar phenomena were then conceptualized. These concepts were further classified and combined into distinct initial categories. Ultimately, 65 open codes emerged, with a subset illustrated in Table 3. It should be noted that some original statements were desensitized due to confidentiality principles governing the interviews.

**Table 3:** The open coding results.

**The raw statements and narratives**	**Initial concept labeling**	**Initial categories**
CF20230711YFJ “We hope that the country will establish only one CMSC in small localities. In reality, however, many counties have two CMSCs, which makes it impossible to form the organizational structure of the CMSCs.”	For small cities, having one CMSC has a more solid organizational structure than having two.	The number of CMSCs determines the overall effectiveness of CMSC construction in the county.
CF20230711YFJ “Without the support of prepaid total medical insurance funds, there is no incentive to construct CMSCs. In many places, they have not actually achieved shared responsibility for overspending or retained surpluses.”	The prepaid total medical insurance funds system provides incentives for the construction of CMSCs.	The medical insurance payment policies provide incentives for the construction of CMSCs.
CF20230711YFJ “The construction of CMSCs requires the support of top county-level officials (county party secretary/county governor), otherwise it is very difficult to promote.”	The top county-level officials support the construction of CMSCs.	The top county-level government promotes the construction of CMSCs.
YZ20230711LYF “Communication between departments is difficult. There is a system of regular meetings, but it has become a mere formality. This is because each department has a different perspective and different interests.”	Difficulty in communication and coordination between departments due to different perspectives and interests.	Difficulty in communication and coordination between departments.

### Axial coding result

**Figure 2:**
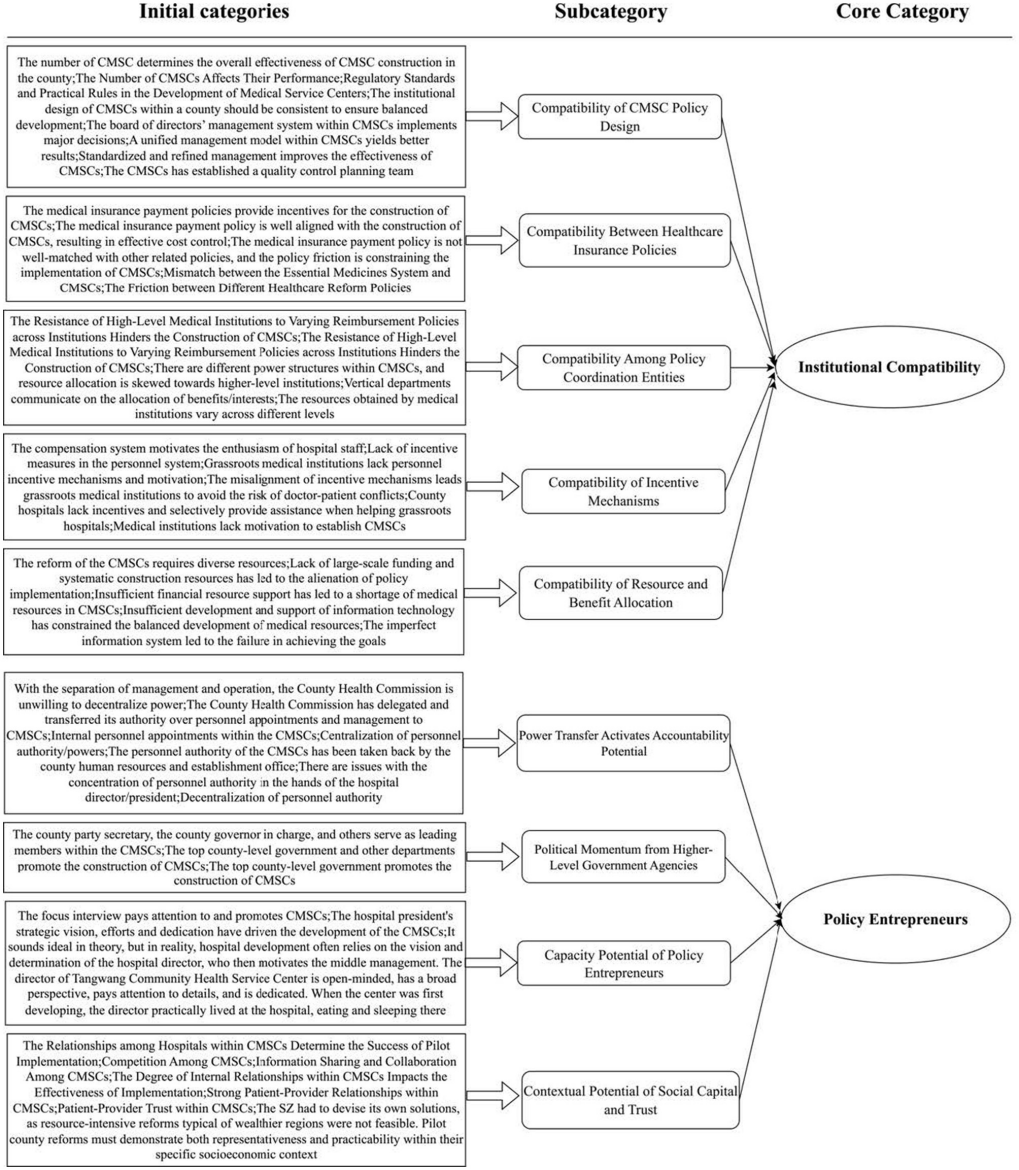
Coding processing.

The study employs axial coding to explore the relationships between the initial categories derived from the data. The primary objective of axial coding is to uncover the psychological intentions and deep-rooted motivations expressed by the participants within these categories. Building upon the open coding process, the study further compares, summarizes, and abstracts the initial categories based on semantic and similarity relationships. This iterative process ultimately yields 9 subcategories and 2 core categories, encompassing various aspects of the pilot management and policy implementation, such as organizational structure and institutional design, incentive mechanisms, power structure adjustment, political influence, policy entrepreneurs, and social network collaboration. The specific coding results, including the categories and subcategories, are presented in Figure 2 of this article.

### Selective coding

Based on the selective coding, the final step of the grounded theory methodology, this study aimed to uncover the core category and validate the relationships between the core category and the main categories. By analyzing policy pilot along two core dimensions-institutional compatibility and policy potential energy-we identify four distinct outcomes of policy pilots: upscaling policy pilot, adjustment policy pilot, suspended policy pilot, autonomous policy pilot.

**Figure 3:**
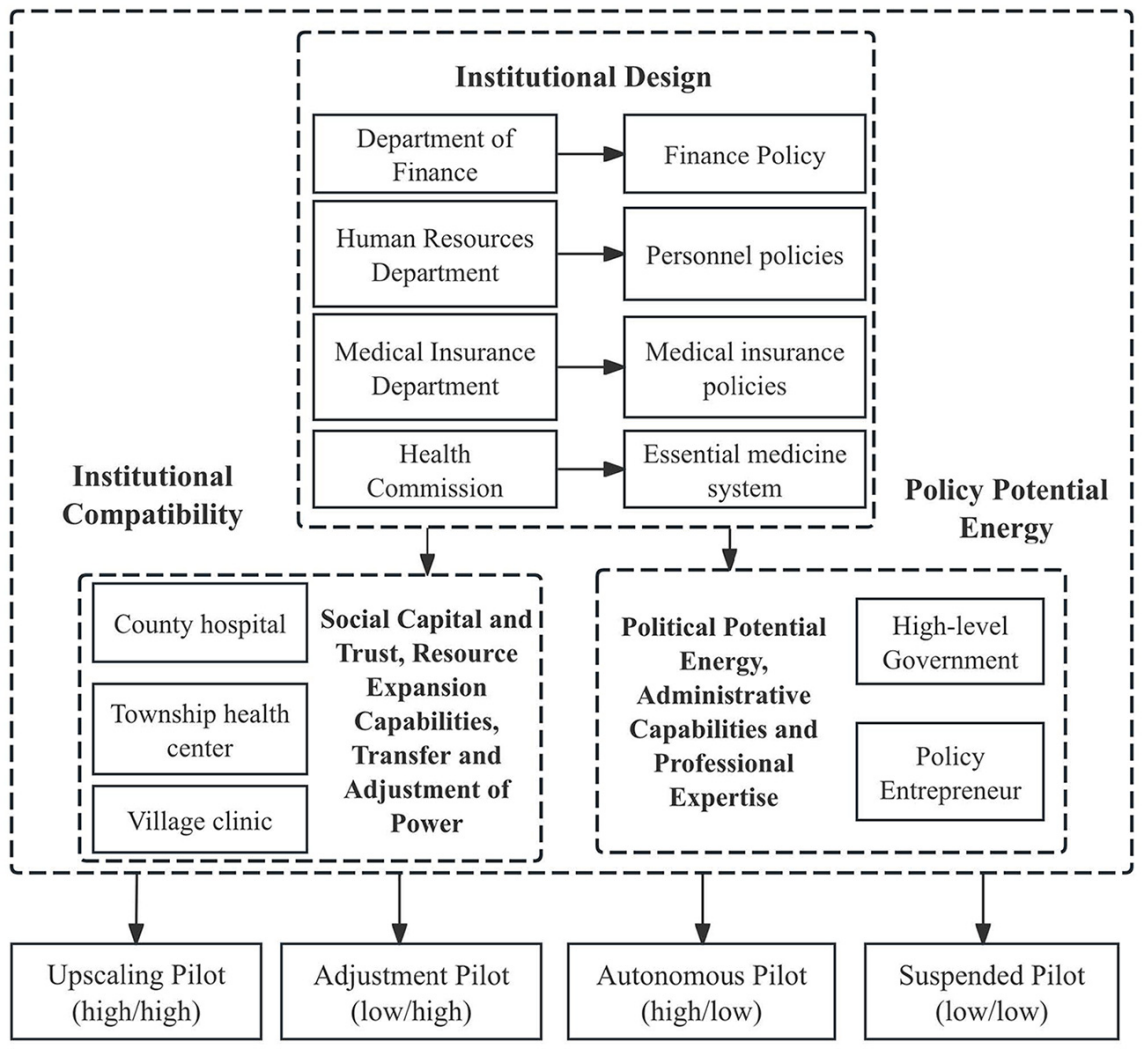
Pilot management and pilot typologies.

As shown in Figure 3, the institutional design of the County Medical Service Community (CMSC) pilot involves multiple governmental departments, with each responsible for formulating policies that collectively shape the governance framework of the CMSC. The Department of Finance develops finance policies, the Human Resources Department formulates personnel policies, the Medical Insurance Department issues medical insurance policies, and the Health Commission establishes the essential medicine system. These policy domains form the institutional foundation upon which the CMSC operates.

Grounded in this institutional design, the CMSC pilot's institutional compatibility can be understood through five key subcategories identified in the coding results: the compatibility of CMSC policy design, compatibility between healthcare insurance policies, compatibility among policy coordination entities, compatibility of incentive mechanisms, and compatibility of resource and benefit allocation. Together, these subcategories constitute the core category of Institutional Compatibility, reflecting how well the pilot aligns with and integrates existing institutional arrangements. Similarly, the pilot's policy potential energy derives from four additional subcategories: power transfer activates accountability potential, political momentum from higher-level government agencies, the capacity potential of policy entrepreneurs, and the contextual potential of social capital and trust. These components collectively form the core category of Policy Entrepreneurs, highlighting the pivotal role of actor capacity, political support, and social embeddedness in advancing pilot reform.

The interaction between Institutional Compatibility and Policy Entrepreneurs ultimately shapes the trajectory of CMSC pilot outcomes, resulting in four distinct types of pilot development: upscaling, adjustment, autonomy, and suspension. These pathways reflect the varying combinations of institutional alignment and policy potential that determine whether and how pilot reforms progress.

## The core theoretical category

### Institutional compatibility

#### Compatibility of CMSC policy design

The institutional design of CMSCs profoundly shapes the coherence, stability, and long-term effectiveness of county-level healthcare integration. A clear and rationalized policy framework helps ensure that the number, structure, and governance functions of CMSCs correspond to local demographic, geographic, and health service capacity conditions. In several pilot counties, respondents emphasized that the absence of unified design principles often results in either redundant establishment of multiple CMSCs or overly centralized structures that do not match actual service needs. One respondent noted the consequences of unclear institutional design: “The number of CMSCs we set up initially was not based on any clear standard… later we realized that too many centers made coordination difficult, and each one fought for resources without a common direction” (HZ20230908LY).

A unified governance model—supported by clear rules on decision-making, performance evaluation, and quality control—has proven essential for CMSCs to function as integrated entities rather than loose hospital networks. Counties that adopted standardized CMSC governance structures reported smoother coordination and higher service quality. “Once we established a board of directors and a unified management office, the hospitals under the CMSC finally followed the same policies. Before that, every hospital did things differently” (TM20230711WXY). Where policy design remained ambiguous, CMSCs encountered role confusion, overlapping responsibilities, and inconsistent management practices. Overall, institutional compatibility requires design principles that are standardized yet sufficiently flexible to adapt to local needs.

#### Compatibility between healthcare insurance policies

Healthcare insurance policies, especially payment mechanisms, constitute a major driver of CMSC behavior. In many pilot counties, mismatches between CMSC goals and reimbursement rules have resulted in fragmented implementation. Respondents commonly reported that medical insurance payment reform did not progress at the same pace as CMSC organizational reform, weakening incentives for hospitals to participate actively. “The CMSC is supposed to integrate county-township-village care, but our medical insurance still reimburses each hospital separately. That means every hospital tries to maximize its own revenue instead of thinking as a group” (XA20230912MY). In counties where medical insurance payment was better aligned with CMSC goals—such as through global budget management or cross- institutional cost control—reform implementation progressed faster and with greater cooperation among institutions. “Our medical insurance payment reform simply didn't match the CMSC design. Policies contradicted each other, so doctors didn't know which rules to follow. This seriously affected implementation” (GZ20230914HX).

#### Compatibility among policy coordination entities

CMSC construction requires coordinated action across administrative entities, yet interdepartmental coordination remains one of the greatest institutional challenges. According to the initial categories, “The Resistance of High-Level Medical Institutions to Varying Reimbursement Policies across Institutions Hinders the Construction of CMSCs.” Resistance emerges when hospitals perceive potential loss of financial benefits or autonomy, making it difficult for county governments to mobilize cooperation. Moreover, the image coding reveals systemic coordination obstacles: “The Resistance of Upper-Level Medical Institutions to Varying Reimbursement Policies and Approval Processes Across Institutions Hinders the Construction of CMSCs.” County-level integrated systems thus confront vertical administrative barriers, limiting the alignment of tasks between hospitals and government agencies.

Horizontal fragmentation also creates problems. Respondents indicated that medical institutions often act independently, with limited information exchange: “Medical institutions do not communicate at all; information sharing varies across different levels.” This lack of coordinated information systems obstructs joint diagnosis, referral, and case management. Institutional misalignment is further reflected in resource management: “The resources and benefits obtained by medical institutions are slow to transfer to lower levels,” causing CMSCs to struggle with downward resource redistribution. Without effective interdepartmental coordination, CMSCs cannot fully achieve integration, even when policies exist on paper.

#### Compatibility of incentive mechanisms

The compatibility of incentive mechanisms is central to motivating participation in CMSC construction. The initial coding identifies multiple factors that weaken incentives among hospitals and medical staff. One key issue is insufficient compensation: “The compensation system motivates the enthusiasm of hospital staff; lack of incentive mechanisms leads to insufficient motivation.” When CMSC reforms introduce new responsibilities without corresponding rewards, motivation declines. Another significant barrier lies in personnel concerns and interest conflicts. Respondents indicated that county hospitals sometimes selectively withhold support from primary care institutions to avoid internal tension: “County hospitals lack incentives and selectively provide assistance when helping grassroots hospitals to avoid the risk of doctor-patient conflicts.” Such selective cooperation obstructs integrated service delivery.

Primary care institutions face similar challenges. Due to inadequate staffing and limited career development pathways, grassroots providers lack internal incentives to engage actively in CMSCs: “Grassroots medical institutions lack motivation to establish CMSCs due to personnel shortages and insufficient support.” These issues underscore that incompatible incentive structures undermine collaboration, inhibit downward resource flow, and weaken the organizational cohesion required for CMSC reform. Strengthening financial, professional, and institutional incentives is therefore critical to supporting integrated county-level healthcare governance.

#### Compatibility of recourse and benefit allocation

Pilot management in healthcare reform is inherently complex and requires careful attention to the allocation of financial, human, technological, and policy resources (46). The effectiveness of pilot initiatives largely depends on the strategic distribution of these limited resources across competing priorities. However, resource asymmetry frequently leads to uneven development, with some components of reform receiving disproportionate attention while others remain underdeveloped. The capacity of local governments to mobilize external resources thus plays a decisive role in shaping their ability to coordinate diverse actors in CMSC construction.

For example, LJ County demonstrated strong fiscal commitment to reform despite limited local resources: “They allocated 10.5 million yuan specifically for healthcare reform and medical consortium development; 3 million yuan for external expert consultation; matching funds of 1.5 million yuan each from two groups for complex category III and IV surgeries, totaling 4.5 million yuan; 6 million yuan for village doctor support; and 1.5 million yuan for talent cultivation and research. LJ County also dispatches expert teams for six-month residencies at branch hospitals to enhance diagnostic and treatment capacity” (LJ20230914WZ).

Conversely, limited access to external resources constrains county governments' ability to mobilize stakeholders and address local healthcare needs. As another respondent observed, “Primary- level hospitals struggle to manage 50+ common and chronic diseases due to inadequate hardware and software capabilities. Substantial support from tertiary hospital experts (3–5 specialists) is necessary to bolster primary care. National regional medical centers remain concentrated in major cities, and resources do not flow downward. Consequently, relying solely on county and township-level tiered diagnosis and treatment is insufficient” (LJ20230915WDL).

Given the dynamic and long-term nature of healthcare reform, continuous evaluation and flexible adjustment of resource allocation strategies are essential. Regular assessments help identify bottlenecks, improve prioritization, and ensure that resource deployment aligns with evolving reform demands. Such adaptive management enhances the likelihood that pilot innovations can be scaled and sustained across broader reform contexts.

### The core pilot actors and their policy potential energy in the CMSCs

#### Power transfer activates accountability potential

The construction of CMSCs requires a complex redistribution of authority across key administrative departments—including finance, health, medical insurance, and human resources. Achieving effective reform thus hinges on appropriately calibrating this power reconfiguration to ensure coordination, accountability, and efficient implementation. Some pilot counties have demonstrated positive outcomes by adapting power structures to local conditions, thereby accommodating variation in administrative capacity and healthcare system characteristics.

For instance, DQ County implemented a systematic redistribution of authority: “The Health Commission shifted from directly managing personnel, finances, and operations to focusing on planning, policymaking, and regulatory oversight. The Commission issued the ‘List of Rights and Responsibilities for the DQ County Health and Family Planning Commission and DQ County Health Care Group,' delineating responsibilities in personnel management, information systems, financial auditing, healthcare reform, scientific education, public health, maternal and child health, and pharmaceutical and medical device management” (DQ20230505WXL).

However, comparative evidence indicates that inadequate or imbalanced power adjustments often hinder reform implementation. Improper decentralization, excessive concentration, or poorly designed delegation mechanisms can undermine stakeholder incentives and weaken reform motivation within CMSCs. As one hospital director reflected, “Originally I was the director of a hospital, but after the superiors sent someone, they had authority over me, which made me feel restricted and uncomfortable” (YZ20230711LYF). Another interviewee noted issues related to personnel authority: “The human resources department reclaimed personnel power. Recruitment exams make hiring more standardized, and giving too much power to the director is not ideal. During one investigation, a hospital had recruited many non-staff without informing the district health committee” (YZ20230711LYF).

These cases illustrate that the transfer and adjustment of power within CMSCs can either facilitate or impede reform, depending on whether the redistribution aligns with local administrative realities and institutional interests. Effective CMSC development therefore requires transparent, balanced, and context-sensitive power arrangements that promote cooperation while preventing administrative conflict and accountability gaps.

#### Political momentum from higher-level government agencies

In the management of CMSC pilots in China, the political potential energy concentrated within county-level party committees and governments constitutes a fundamental driving force for advancing reform. Since the reform and opening-up, the operation of China's political-administrative system has emphasized the interdependence of the state, society, the Party, and the bureaucratic apparatus, treating them as a collective action alliance. Within this system, county governments possess authority that extends across departmental and organizational boundaries. Their capacity to mobilize diverse actors, strengthen interdepartmental coordination, and integrate decision-making processes is therefore essential for enabling effective and innovative governance at the county level. This mode of operation permeates all arenas of county governance, including the establishment and functioning of CMSCs.

Within China's healthcare reform experiments, the CMSC is not propelled by a single institution but emerges from the collective efforts of multiple stakeholders. Among these actors, county-level top officials play an especially decisive role. As core agents of reform, their political authority and governance capabilities fundamentally shape the degree of coordination achieved among participating departments and institutions (47). By leveraging political potential energy, these leaders mobilize key actors, align their interests with county-level health reform priorities, and reinforce the overall direction of the CMSC pilot.

Empirical evidence demonstrates that successful pilots frequently rely on strong engagement from high-level local leaders, who actively transfer authority to CMSCs and embed reform responsibilities within existing administrative hierarchies. As one interviewee described: “The organizational structure of the CMSC has the county party secretary as the director of the health commission, the county governor as the first deputy director, and three other deputy directors from the medical, health insurance, and health commission departments. There is also a dedicated deputy director responsible for the CMSC, with committee members below. At the township and village levels, responsibility mainly falls on township heads. The entire organizational support is driven from the top; otherwise, it would not be possible to implement” (LJ20230914WZ).

In contrast, insufficient attention from senior officials undermines reform momentum and exacerbates vertical collective-action dilemmas. As another respondent noted: “Documents issued jointly by multiple departments are more effective than those issued solely by the municipal party committee or government, because many departments simply do not implement some documents, making real policy implementation very difficult” (YZ20230711LYF).

Higher-level government departments also play a significant role in mobilizing and coordinating key stakeholders, such as medical insurance bureaus and health commissions. This top–down coordination mechanism helps to overcome entrenched departmental interests, rectify power imbalances, and create institutional conditions conducive to policy innovation and effective implementation (47). Through their institutional authority and resource endowments, higher-level authorities can: (1) establish frameworks for cross-departmental collaboration; (2) support relatively weaker departments to ensure they can participate meaningfully in reform; and (3) develop incentive mechanisms that encourage active engagement across agencies. Such strategic intervention mitigates the persistent problem of fragmented authority in policy implementation and strengthens the institutional foundation for advancing healthcare reform.

The political capital possessed by higher-level government departments is thus critical for persuading and integrating actors—particularly medical insurance departments and health commissions—into the management of pilot reforms. Their involvement not only enhances vertical coordination but also increases the likelihood of achieving more coherent, sustainable, and scalable reform outcomes.

#### Capacity potential of policy entrepreneurs

Policy entrepreneurs constitute a critical group of actors in the development and management of CMSCs, owing to the highly specialized and institutionally complex nature of healthcare reform. Situated at the intersection of government authorities and healthcare institutions, these entrepreneurs play a bridging role that connects administrative mandates with professional practice. Upwardly, they engage actively with government agencies to secure essential support in funding, personnel, and policy authorization; downwardly, they design management mechanisms and operational rules that integrate diverse participants and coordinate resource allocation. High levels of elite cohesion among these actors strengthen their mobilization capacity, allowing for more flexible and strategic selection of organizational approaches. This, in turn, contributes to more efficient resource distribution and incentive alignment, thereby enhancing the county government's ability to achieve CMSC reform objectives. As one interviewee noted: “It sounds ideal in theory, but in reality, hospital development often relies on the vision and determination of the hospital director, who then motivates the middle management. The director of Tangwang Community Health Service Center is open-minded, has a broad perspective, pays attention to details, and is dedicated. When the center was first developing, the director practically lived at the hospital, eating and sleeping there” (YZ20230712LYF).

Unlike the governance of routine administrative matters at the county level, the healthcare sector—encompassing medical education, personnel training, employment pathways, and professional development—operates under a distinct logic of professionalism characterized by medical authority, technical autonomy, and relative institutional independence. As direct providers of medical services, healthcare institutions hold a structural advantage in service delivery and play an indispensable role in CMSC construction. At the current stage of reform, building effective CMSCs requires the alignment and compatibility of administrative and professional logics. This necessitates that leading hospitals and hospital-based policy entrepreneurs, acting as delegated agents, translate the decision-making intentions of county-level party committees and governments into concrete organizational practices. They are also expected to assume the risks associated with policy innovation and promote implementation with a strong sense of professional responsibility and institutional commitment.

As one respondent described: “The party secretary and our hospital director led our relevant middle-level personnel and cadres in conducting a major investigation. They effectively linked our policies with the local government, town government, and village government, and comprehensively aligned our methods and ideas with theirs. If we had relied solely on our doctors in white coats to do this, it might have been quite difficult” (LJ20230914WXX).

By leveraging their expertise and professional judgment, policy entrepreneurs within leading hospitals are able to secure additional resources and institutional support for CMSC construction, thereby facilitating the smooth implementation of county-level healthcare reform. Their combined administrative responsiveness and professional competence thus constitute a central mechanism through which CMSC pilots are advanced and sustained.

#### Contextual potential of social capital and trust

In the face of institutional change and environmental uncertainty, social networks provide CMSCs with critical adaptive capacity. Robust inter-organizational networks facilitate information exchange, knowledge sharing, and collaborative problem-solving across medical institutions, thereby strengthening the internal coordination mechanisms of CMSCs. The accumulation of social capital nurtures trust-based relationships among stakeholders, reduces cooperation-related uncertainties, and enhances incentives for sustained collective action.

Within counties, multiple CMSCs may coexist, forming relationships that are collaborative in principle yet competitive in practice. As one interviewee explained, “In our county, the two CMSCs have a good, united relationship since the director of the Chinese medicine hospital used to be the secretary of our hospital. But in some counties with up to four CMSCs, there is constant bickering” (HZ20230908YS). Another interviewee highlighted the risks of siloed operations: “The two CMSCs don't share patient information, so they are unaware of treatment situations in each other's jurisdictions. They act independently, content with just meeting standards” (TM20230711YXY).

Patients, as the ultimate service recipients, also constitute a vital dimension of social capital. Building trust with the public is essential for the effective functioning of CMSCs. Strong patient trust facilitates service uptake, strengthens the accountability of healthcare providers, and enhances intrinsic motivation for performance improvement within the collaborative network. As one participant noted, “We arranged for postgraduate students from universities to provide consultations in the community, forming a trust mechanism between patients and doctors. This project later became a nationwide reform case study” (XA20230912MY).

Overall, social capital—both horizontally among institutions and vertically between CMSCs and patients—forms a foundational institutional resource that supports cooperation, reduces coordination costs, and improves the performance of pilot management.

## Extended analysis-four outcomes of policy pilots

By analyzing policy pilots through two core dimensions—institutional compatibility and policy potential energy—this study extends existing theories of policy implementation and develops a new analytical framework for explaining variations in pilot outcomes. This approach builds on, but moves beyond, the foundational insights of Pressman and Wildavsky's (23) classic implementation analysis and Matland's (26) ambiguity–conflict model. Whereas, Pressman and Wildavsky emphasized the cumulative challenges of inter-organizational coordination, and Matland theorized how ambiguity and conflict shape implementation processes, our framework introduces a more explicitly institutional and dynamic understanding of pilot governance, capturing how structural alignment and political–administrative momentum interacts to shape the trajectories of contemporary policy experiments. In contrast to Matland's focus on interpretive ambiguity and stakeholder conflict, the “institutional compatibility × policy potential energy” matrix should be understood as an extension rather than a simple adaptation: it translates the logic of ambiguity and conflict into empirically grounded dimensions relevant to iterative, multilevel policy experimentation—namely, the coherence of institutional arrangements and the strength of political, administrative, and social forces sustaining pilot evolution.

This analytical contribution also differs from existing China-focused studies on pilot reforms, such as Song and Li's (1) work on the management of NRCS pilots and He et al. (3). and Li and Song (9) on hospital reform pilots. These studies highlight the importance of political support, administrative capacity, and bureaucratic coordination, but they tend to analyze these factors separately or treat them as background conditions. In contrast, our “driving forces” —Institutional compatibility—reflects the alignment of CMSC policy design, healthcare insurance payment rules, interdepartmental coordination, incentive structures, and resource–benefit allocation. They represent a systematic and empirically grounded configuration of the institutional mechanisms that condition whether county-level reforms can operate as integrated systems. On the other dimension, “policy potential energy” synthesizes insights from comparative research on experimentalist governance (29, 48), policy learning (37, 49), and multilevel state capacity, but reworks them into a unified concept that captures the dynamic accumulation of political momentum, redistributive authority, entrepreneurial leadership, and social trust that enables pilots to move beyond their initial design.

**Figure 4:**
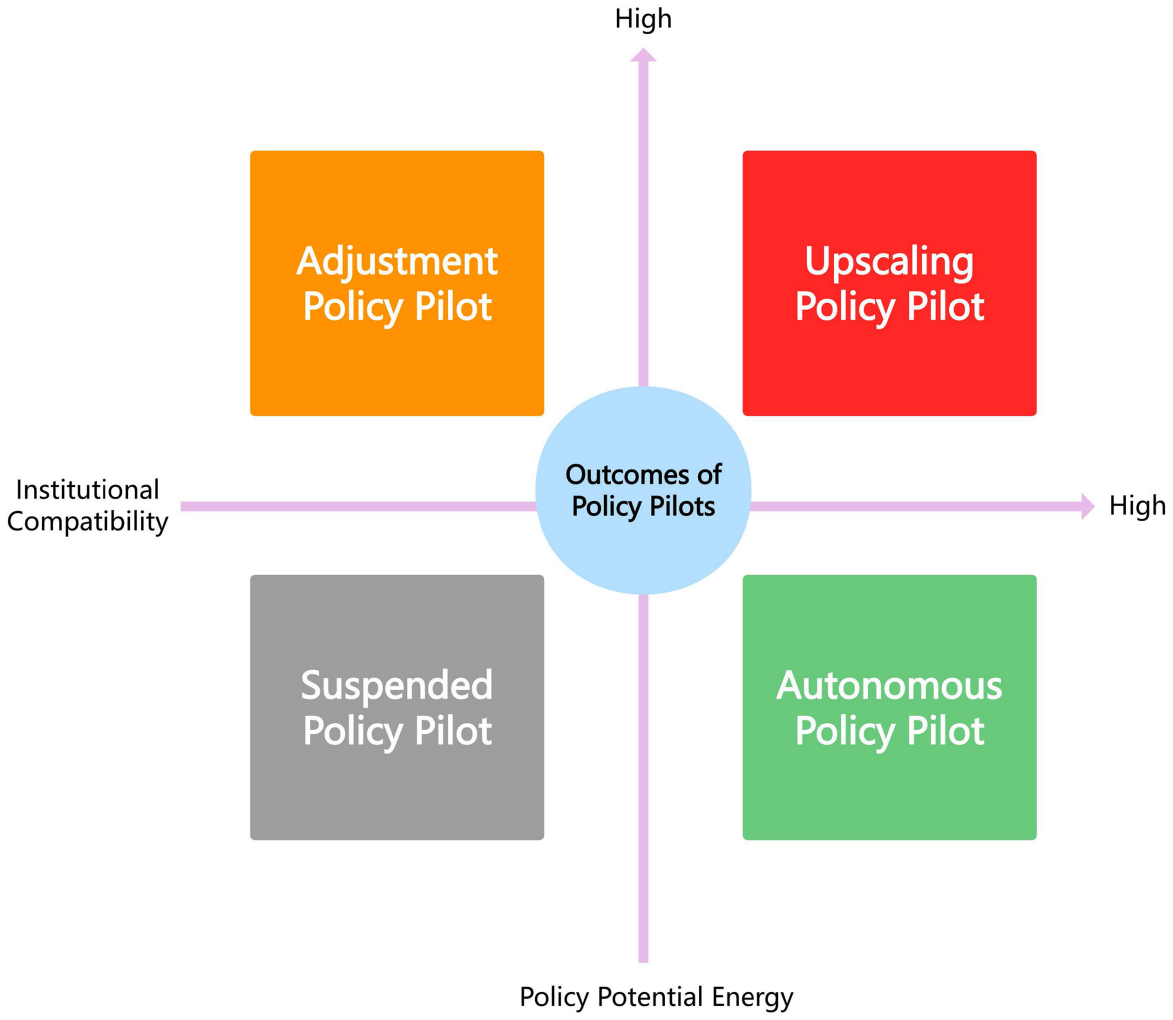
Four distinct outcomes of policy pilots.

Taken together, these two dimensions allow us to identify four distinct types of pilot outcomes—upscaling, adjustment, suspension, and autonomy—as illustrated in Figure 4. This typology contributes to international debates by showing that variation in pilot trajectories cannot be explained solely by capacity, political support, or conflict levels, but by the interaction between institutional alignment and the mobilization of policy potential energy across governance levels. In doing so, the study provides a theoretically grounded and empirically informed framework that enhances comparative understanding of why policy pilots diverge over time, both within China and in broader international contexts.

### Upscaling policy pilot: DQ county

Characterized by high institutional compatibility and high policy potential energy, these pilots are most likely to be adopted on a larger scale. Institutional compatibility refers to the alignment of a pilot's power structures, resource allocation, and interest distribution with the local institutional context and existing healthcare reform policies. This compatibility ensures that the pilot can be integrated into the existing system without causing major disruptions or conflicts (50). High policy potential energy, by contrast, indicates that the pilot is propelled by strong momentum from higher-level government authorities as well as the administrative and reform capacities of policy entrepreneurs (51). This combination of top–down political pressure and bottom–up professional expertise creates a favorable environment for policy adoption and implementation (52). Policy entrepreneurs who can navigate healthcare system complexities and mobilize resources play a crucial role in enabling pilot programs to scale up successfully (53). The synergy between institutional compatibility and policy potential energy minimizes resistance to change, leverages existing structures, and capitalizes on both political momentum and organizational capacity (54). Together, these factors significantly increase the likelihood of scalable and sustainable healthcare reform implementation (55).

The CMSC reform in DQ County illustrates the interaction between institutional compatibility and strong policy momentum in advancing China's healthcare transformation. Institutional compatibility is reflected in the restructuring of power relations and the integration of medical resources. The county established two major consortia linking county hospitals with township health centers, achieving vertical integration. It implemented unified financial management and an innovative medical insurance payment model combining outpatient capitation with DRG-based inpatient payments. These arrangements aligned with existing local institutional conditions and national reform directions. The consortia were granted autonomy in personnel management, performance assessment, and income distribution, enhancing institutional flexibility. Complementing these structural reforms, policy momentum was driven by strong political support, including direct endorsement by the county party secretary at a county-wide health conference. This was reinforced by bottom–up innovation from policy entrepreneurs within the local health bureau. Together, these elements—power restructuring, resource integration, political support, and administrative innovation—demonstrate how institutional compatibility and policy momentum jointly facilitate county-level healthcare reform. This corresponds with Howlett and Rayner's (56) framework emphasizing the importance of structural alignment and dynamic policy processes.

“Our medical consortium reform primarily revolves around five aspects of integration. Firstly, institutional integration: we established two major healthcare groups, consolidating all medical and health resources across the county. Secondly, personnel integration: the consortium has substantial autonomy in human resource management, implementing unified recruitment, training, and deployment. Thirdly, financial integration: we established a centralized operations management department responsible for fiscal budgeting and financial management. Fourthly, information integration: we invested in constructing a unified management platform, achieving consortium-wide unified management. Lastly, resource integration: we restructured and consolidated all resources within the medical consortium, implemented unified procurement, and established shared service centers, significantly enhancing resource utilization efficiency. These measures have substantially improved our operational efficiency and service quality” (DQ20230515WL).

Uvin (57) and Uvin and Jain (58) identify four types of upscaling for pilot innovations: size/coverage, activities, indirect influence, and sustainability. Size/coverage refers to geographical or population expansion. Activities upscaling involves diversifying or integrating new activities. Indirect influence broadens the pilot's political effects on other groups. Sustainability enhancement focuses on resource diversification, external partnerships, managerial improvement, and learning from practice. The sustainability of the DQ reform is evident in its continued evolution. As noted by Vice Governor Cheng Yuechong during his visit to DQ County on October 11, 2022: “DQ has achieved a ‘win-win-win' situation through reform and innovation. Moving forward, it is crucial to accurately grasp the direction and key elements of reform, further explore breakthroughs, and establish standards, thereby paving a new path for province-wide healthcare payment reform.” (DQ20230505LHM) This official endorsement shows that the DQ model not only sustained initial success but continues to evolve, demonstrating the sustainability dimension of upscaling. This ongoing adaptation also reflects indirect influence upscaling, in which the pilot's impact extends beyond its immediate locality (59).

### Autonomous policy pilot: SZ county

Featuring high institutional compatibility but low policy potential energy, these pilots tend to operate independently without broader diffusion. SZ County exemplifies this type. Benefiting from its recent experience in poverty alleviation reforms, SZ demonstrates relatively high institutional compatibility; however, its policy potential energy remains low, as the county primarily relies on self-initiated efforts rather than substantial external resources or political pressure. Following a “three-step” process, SZ integrated three county-level hospitals into a single entity, establishing a unified management structure and optimizing resource allocation. This restructuring aligns with the concept of institutional layering, in which new components are introduced within existing institutional arrangements. The county's eight reform measures—including deepening medical insurance payment reform and strengthening public health management—reflect a comprehensive approach that builds upon existing structures while incorporating innovative elements.

Despite this institutional compatibility, SZ's reform efforts exhibit low policy potential energy compared to resource-rich localities. As a former poverty-stricken county, SZ has relied heavily on endogenous innovation and adaptive strategies, with actors creatively leveraging limited resources to pursue reform goals. As one interviewee noted, “SZ had to devise its own solutions, as resource-intensive reforms typical of wealthier regions were not feasible. Pilot county reforms must demonstrate both representativeness and practicability within their specific socioeconomic context” (SZ20230831GZY).

SZ's “learning by doing” approach further illustrates the importance of policy learning in low-resource settings. “Initially, the People's Hospital and the Traditional Chinese Medicine Hospital separately led the establishment of semi-integrated CMSCs. However, this approach proved ineffective, resulting in significant deficits in the medical insurance fund. After studying the Sanming healthcare reform model, SZ County established a tightly integrated CMSC by consolidating three county-level hospitals into the SZ County General Hospital and integrating pharmaceutical management, medical insurance, and healthcare delivery—the ‘three medical linkages' in Chinese healthcare reform” (SZ20230831HQG).

The SZ case demonstrates that even under conditions of high institutional compatibility but limited policy potential energy, substantial reforms can be achieved through self-driven efforts, creative adaptation, and strategic policy learning. This underscores the need to consider both institutional and resource dimensions when analyzing variation in policy implementation and scaling.

### Adjustment policy pilot: YZ county

With low institutional compatibility but high policy potential energy, these pilots require substantial modification before broader upscaling becomes feasible. The case of YZ offers a compelling example of a pilot characterized by this combination. Low institutional compatibility led to significant divergence from the nationally prescribed CMSC model, yet strong policy potential energy—generated by high-level pressure and capable local policy entrepreneurs—enabled the development of a regionally adapted healthcare system that has produced notable results.

YZ initially attempted to implement the CMSC model as mandated by higher authorities. However, local officials encountered major obstacles due to the low level of institutional compatibility. As the Director of the YZ Health Commission explained: “County hospitals are unwilling to take on generally poor-performing health centers. Firstly, they're a burden; secondly, the support effects are not obvious, and county hospitals have limited resources. From the national perspective, ideally, there should be 2–3 CMSCs per county. Currently in YZ, neither county hospitals nor grassroots hospitals have the motivation to form CMSCs. According to national directives, we need to shift from full institutional coverage to full service coverage. Originally, the state required medical institutions in every township and village, but with an aging population, this model will certainly not develop well” (YZ20230711LBH).

Confronted with these challenges—and under pressure to demonstrate compliance with the national CMSC model—YZ explored an alternative approach better aligned with local conditions. This led to the formation of a regional medical consortium model, which proved more feasible and effective. The Health Commission Director elaborated: “The development of regional medical consortia may be more practical and can accomplish many things. We aim to concentrate medical personnel and community health resources in rural regional centers, strengthening regional leaders. This way, most medical issues can be resolved locally, with referrals to higher-level hospitals only when necessary. For surrounding general health centers, we focus more on developing outpatient and health management functions” (YZ20230711LBH).

Ultimately, YZ's CMSC pilot evolved along two parallel tracks: one designed to satisfy higher-level government requirements, and another adapted to local needs. As a local official explained: “Both models exist in YZ. Many reforms involve personnel and financial adjustments, which are difficult to implement through top-down mandates. Counties can choose which model to follow, explore freely, or even pursue both models.” This dual-track approach reflects “formal compliance and informal adaptation” in China's policy implementation processes.

YZ's experience illustrates how regions with low institutional compatibility but high policy potential energy can navigate tensions between national mandates and local realities. By leveraging local knowledge, administrative capacity, and adaptive strategies, local actors can reinterpret and modify national policies, generating innovative institutional solutions that still achieve core reform objectives.

### Suspended policy pilot: GZ county

Characterized by both low institutional compatibility and low policy potential energy, these pilots are the most likely to be discontinued. The CMSC pilot in GZ exemplifies this scenario. This outcome aligns with May and Winter's (60) notion of “implementation failure,” in which policies falter because of a misalignment between policy design and implementation context, compounded by insufficient political or administrative support.

Low institutional compatibility in GZ is reflected in substantial regional disparities and cultural barriers. As one local health official noted: “The disparity between urban and rural development in the Gannan region is already significant. The public welfare nature of grassroots medical institutions is guaranteed at the municipal level, but safeguard measures at the national and provincial levels remain inadequate.” (GZ20230812ZGX) Integrating external experts into local healthcare institutions has also proven difficult: “Due to differences in culture and customs, it is difficult for outside experts to integrate into the local community and work cohesively. Experts from exporting hospitals often serve as administrative directors or executive presidents in receiving hospitals, which can lead to conflicts with local experts” (GZ20230812ZGX).

The pilot also exhibits low policy potential energy, reflected in shallow implementation, weak reform motivation, and insufficient support. As a respondent explained: “Since the tightly integrated CMSC model is not being implemented very deeply, there is little practical experience regarding integration at township and village levels.” Another added: “Improving grassroots medical service capacity under the current model requires a long process because hospitals have little motivation to promote hierarchical diagnosis and treatment.” (GZ20230812HYS) This corresponds to Matland's (26) description of low policy salience, which undermines implementation.

Support for reform is similarly limited. One interviewee observed: “Previous models often became mere formalities and failed to provide effective support. Full integration remains challenging, requiring the removal of policy and communication barriers between government and hospitals, between prefecture-level and county-level hospitals, and between provincial and local institutions” (GZ20230812QW). Given these conditions, the CMSC pilot in GZ ultimately entered a suspended state, with no substantial progress observed during the investigation.

## Conclusion

This study investigated the implementation dynamics of China's CMSC pilots through extensive fieldwork and grounded theory analysis. By identifying institutional compatibility and policy potential energy as the two central forces shaping pilot evolution, the study demonstrates that pilot outcomes emerge not only from policy design but from the interaction between structural alignment and the mobilization of political, administrative, and social resources. These interactions generate four distinct trajectories—upscaling, adjustment, autonomy, and suspension—offering a more nuanced understanding of variation in pilot outcomes.

Theoretically, the study strengthens international scholarship on policy experimentation and implementation in three ways. First, it links grounded, field-derived mechanisms to classical implementation theories, extending insights from Pressman and Wildavsky's coordination framework and Matland's ambiguity–conflict model to contemporary experimental governance contexts. Second, by drawing on comparative work on experimentalist governance and policy learning, the study shows how iterative adaptation, inter-organizational coordination, and actor capacity jointly shape pilot performance. Third, the typology of four pilot trajectories moves beyond binary assumptions of success vs. failure, providing a more differentiated conceptualization of how experimental policies evolve under varying institutional and political conditions.

Practically, the findings highlight several implications for healthcare reform governance. Improving institutional compatibility requires greater coherence across policy domains—including medical insurance payment rules, personnel systems, fiscal management, and information integration. At the same time, enhancing policy potential energy depends on sustained political commitment, capable policy entrepreneurs, and trust-based collaborative networks among institutions. The proposed typology offers a diagnostic tool that can help policymakers anticipate implementation bottlenecks, tailor intervention strategies, and provide differentiated support to counties facing distinct challenges. These insights can guide future rounds of CMSC reform as well as other complex policy experiments that require multilevel coordination.

Several limitations should be noted. First, although the research provides valuable insights into healthcare reform pilot dynamics, the generalizability of the findings to other policy domains may be limited. Future studies could examine the applicability of this framework in other contexts. Second, the study focuses primarily on short-term and intermediate outcomes; longitudinal research would help clarify the long-term impacts and sustainability of different pilot trajectories. Finally, although key driving forces and outcomes were identified, further research is needed to explore the causal mechanisms linking these elements. Mixed-method approaches or comparative case studies could deepen understanding of these relationships.

## Data Availability

The original contributions presented in the study are included in the article/supplementary material, further inquiries can be directed to the corresponding author.
